# Cardiometabolic comorbidities and cardiovascular events in “non-functioning” adrenal incidentalomas: a systematic review and meta-analysis

**DOI:** 10.1007/s40618-024-02440-0

**Published:** 2024-09-30

**Authors:** Vittoria Favero, Chiara Parazzoli, Davide Paolo Bernasconi, Iacopo Chiodini

**Affiliations:** 1 Department of Medical Biotechnology and Translational Medicine, University of Milan, Milan, 20100 Italy; 2 Unit of Endocrinology, ASST Grande Ospedale Metropolitano Niguarda, Piazza Ospedale Maggiore 3, Milan, 20162 Italy; 3 Bicocca Bioinformatics Biostatistics and Bioimaging B4 Center, Department of Medicine and Surgery, University of Milano-Bicocca, Monza, Italy; 4 Unit of Clinical Research and Innovation, ASST Grande Ospedale Metropolitano Niguarda, Milan, Italy

**Keywords:** Adrenal incidentalomas, Hypertension, Diabetes, Dyslipidaemia, Metabolic syndrome cardiovascular events

## Abstract

**Objective:**

Recent studies investigated the prevalence of arterial hypertension (AH), diabetes mellitus (DM) and/or prediabetes, dyslipidemia (DL), metabolic syndrome (MS) and cardiovascular events (CVE) in patients with non-functioning adrenal incidentalomas (NFAI). We aimed to investigate the available literature to determine the prevalence of AH, DM, DM and/or prediabetes (Composite DM, C-DM), DL, MS and CVE in patients with NFAI as compared to patients without adrenal incidentalomas (AI).

**Design:**

Systematic review and meta-analysis.

**Methods:**

A meta-analysis was performed using studies that evaluated the prevalence of AH, DM, C-DM, DL, MS and CVE in patients with NFAI versus matched subjects without AI. A random-effects model (DerSimonian and Laird) was used to calculate the pooled odds ratio (OR) and 95% Confidence Interval (95%CI) for each outcome.

**Results:**

Among the 36 available studies, 19 studies provided the necessary data (4716 subjects, mean age 57.6 ± 4.6). The association between AH, DM, C-DM, DL, MS and CVE was reported in 18 (4546 subjects), 7 (1743 subjects), 5 (4315 subjects), 11 (3820 subjects), 8 (1170 subjects) and 5 (2972 subjects), respectively. The presence of NFAI was associated with AH (OR 1.87, 95%CI 1.39–2.51), C-DM (OR 2.04, 95%CI 1.70–2.45) and MS (OR 2.89, 95%CI 1.93–4.32), but not with DM, DL and CVE.

**Conclusions:**

Patients with NFAI have higher prevalence of AH, C-DM and MS than control subjects without NFAI.

**Supplementary Information:**

The online version contains supplementary material available at 10.1007/s40618-024-02440-0.

## Introduction

Recently, an increasing number of studies have been focused on the clinical importance of the incidentally discovered adrenal masses (adrenal incidentalomas, AI). This growing interest in AI is due, firstly, to the relevant AI prevalence in the general population, which is estimated to reach 7% in individuals over 60 years of age [1, 2]. Secondly, in about half of patients with AI a condition of mild autonomous cortisol secretion (MACS) could be present, which, though asymptomatic, is associated with a higher risk of diabetes mellitus (DM), arterial hypertension (AH), cardiovascular events (CVE) and even mortality [3–9]. Finally, the evidence that DM and AH control generally improves after the recovery from MACS by adrenalectomy [2, 10–12] has further increased the interest on AI and MACS.

On the other hand, even more recently, sparse data have suggested that, as compared with patients without AI, even patients with AI but without MACS (so called “non-functioning” AI, NFAI), may be at higher risk of DM, AH and CVE [13–32]. The idea that NFAI can produce a certain amount of excess cortisol is sustained by data showing that adrenalectomy seems to improve blood pressure and glycometabolic control even in some patients with NFAI [33] and that these patients may be at risk of post-surgical hypocortisolism after the removal of the adrenal mass [34]. Finally, some studies have suggested that the mortality risk is increased in patients with NFAI [32] and that the extent of this increase is similar to the one described in subjects with MACS [35].

However, the risk of cardiometabolic comorbidities in patients with NFAI is still debated, since, so far, most of the available studies have compared patients with NFAI with patients with MACS, with the formers being considered a control group without cortisol excess [4, 6, 36, 37].

Thus, the aim of the present study was to review the available studies evaluating the prevalence of AH, DM or prediabetes (pre-DM), dyslipidemia (DL), obesity (OB), metabolic syndrome (MS) and CVE in patients with NFAI, as compared with subjects without AI and to perform a meta-analysis assessing the risk of patients with NFAI to have AH, DM or pre-DM, DL, MS and CVE.

## Methods

This study, registered on PROSPERO (ID 544820), was performed following the Preferred Reporting Items for Systematic reviews and Meta-Analyses statement [38].

### Search strategy

**Fig. 1:**
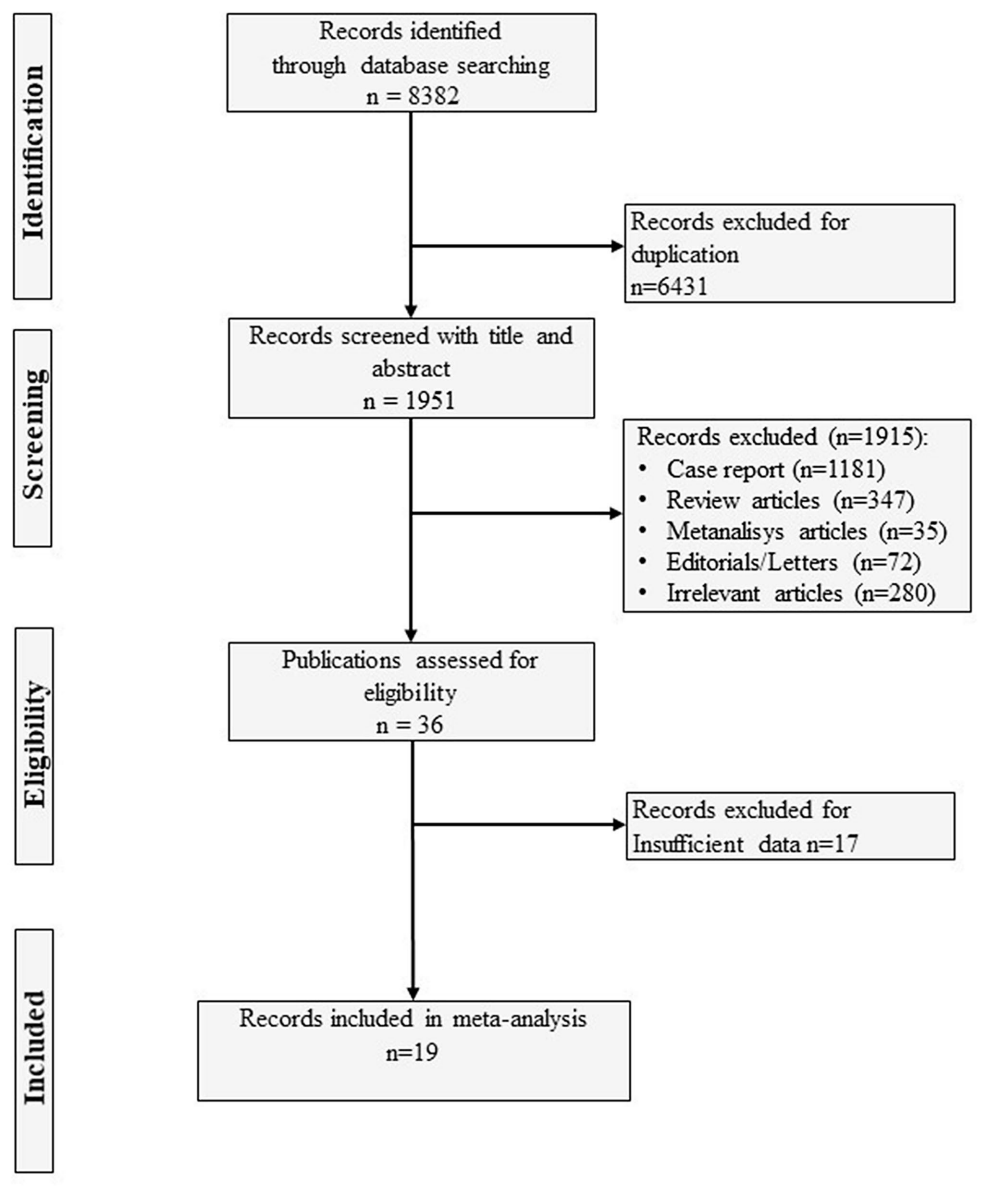
Study selection process. Footnotes: PubMed, Web of Science and Scopus were searched between January 1990 and March 2024 using the following key words: “adrenal incidentalomas, AI, adrenal adenomas, non-functioning adrenal incidentalomas, non-functioning adrenal adenomas, subclinical hypercortisolism, subclinical Cushing’s syndrome, hidden hypercortisolism, mild autonomous cortisol secretion, less severe hypercortisolism, mild hypercortisolism”. A further analysis of the reference lists of the eligible articles was performed to find out other additional publications The Mendeley Desktop application (version 2.112.0, Mendeley Ltd) was used to remove the duplicates and apply the inclusion criteria

Two independent reviewers (V.F. and C.P.) independently reviewed the English literature, screened titles and abstracts and examined the full text of potentially relevant studies. Discordances were resolved by a third reviewer (I.C.). PubMed, Web of Science and Scopus were searched between January 1990 and March 2024 using the following keywords and medical subject headings (MeSH): “adrenal incidentalomas, AI, adrenal adenomas, non-functioning adrenal incidentalomas, non-functioning adrenal adenomas, subclinical hypercortisolism, subclinical Cushing’s syndrome, hidden hypercortisolism, mild autonomous cortisol secretion, less severe hypercortisolism, mild hypercortisolism” (Fig. 1). A further analysis of the reference lists of the eligible articles was performed to find out other additional publications.

The Mendeley Desktop application (version 2.112.0, Mendeley Ltd) was used to remove the duplicates and apply the inclusion criteria.

### Study’s selection

We included only case-control studies (observational, prospective, or retrospective) that evaluated adult patients (aged ≥ 18 years) with NFAI and matched control subjects without AI and other adrenal diseases. All studies had to base the definition of NFAI on the criteria suggested by the European Society Guidelines [39]: (i) a serendipitously discovered adrenal mass above 1 cm in size diagnosed by imaging (computed tomography or magnetic nuclear resonance) performed for unrelated disorder; (ii) no signs or symptoms of hypercortisolism; (iii) no use of glucocorticoids during at least the last 3 months; (iv) cortisol levels after 1-mg overnight dexamethasone suppression test (F-1mgDST) ≤ 1.8 µg/dL (50 nmol/L).

All case series, case reports studies and studies comparing patients with NFAI with patients with MACS but without a matched control group of subjects without AI were excluded. We also excluded studies on patients with NFAI which included subjects with a F-1mgDST > 1.8 µg/dL or using other criteria for ruling out cortisol hypersecretion. Studies including patients with AI and without MACS but including patients with AI and with catecholamines, sex hormones and mineralocorticoids hypersecretion were considered ineligible. Studies including patients with adrenocortical carcinomas and adrenal metastasis were also excluded. Finally, we also excluded those studies in which a control group of individuals not affected by AI was not included (Fig. 1).

### Study outcomes

The predefined primary outcomes were the prevalence of AH, severe AH (i.e. resistant AH or AH treated with ≥ 3 drugs), DM, pre-DM (i.e. impairing fasting glucose and/or glucose intolerance), DL, OB, MS and CVE in patients with NFAI compared to controls without AI.

We considered valid the definitions of AH, severe AH, DM, pre-DM, DL, OB, and MS according to the current clinical practice recommendations [40–43]. The reported prevalence of the following CVE was considered: myocardial infarction, ischemic stroke, transient ischemic attack, angina pectoris, pulmonary embolism, intracerebral hemorrhage, and peripheral artery disease.

### Data extraction

Each study was searched by two authors (V.F. and C.P.) to extract the following data from patients and controls, when available: authors and study location, year(s), study design, sample size, mean age, percentage of male patients, ethnicity, type and prevalence of the outcomes (i.e. AH, severe AH, DM, pre-DM, OB, DL, MS and CVE), association estimate (odds ratios [ORs] and 95% confidence intervals [CIs]), F-1mgDST mean levels, and use of adjustments for the association estimate. In the presence of studies with zero-cell counts, we added a fixed value equal to 0.5 to all cells of the study to estimate the raw OR.

### Quality assessment

Two investigators (V.F. and C.P.) performed both the quality assessment of individual studies and the overall quality of evidence. The modified Newcastle–Ottawa Scale was used to assess the quality of the studies (www.ohri.ca/programs/clinical_epidemiology/oxford.asp).

The following items were evaluated: study sample, selection criteria of patients with NFAI (based on F-1mgDST), comparability (whether patients with NFAI and controls were matched for age, sex, and body mass index, BMI) and outcomes definitions (i.e. how AH, severe AH, DM, Pre-DM, DL, OB, MS and CVE were defined).

### Data synthesis and statistical analysis

We conducted a meta-analysis of all eligible studies and obtained the pooled estimate in patients with NFAI as compared to patients without NFAI separately for AH, severe AH, DM, Pre-DM, DL, OB, MS and CVE) (primary outcomes).

All analyses were performed with R version 4.0.3 (R Foundation for Statistical Computing, Vienna, Austria). Random-effects meta-analysis has been performed by first deriving an estimate of the between-study variation and heterogeneity. Subsequently, these results have been used for combining results (i.e. for estimating the effect) and for developing the figure of primary interest.

The DerSimonian and Laird method, the conventionally used approach for random effects meta-analysis [44] has been used for calculating the association estimates (odds ratio, OR) and their 95% interval of confidence (95% CI). The heterogeneity between studies was quantified using I^2^ and τ^2^ statistics.

We conducted an analysis using Funnel Plots and Egger Test in order to evaluate the presence of possible publication bias and implemented an influence analysis with the leave-one-out method (omitting one study at a time) to investigate the impact of each study-specific association estimate on the pooled OR. Finally, for outcomes with a positive association, we conducted a meta-regression analysis to assess the impact of several covariates (including age, gender and BMI) on the OR, accounting also for the size of each study. P-value lower than < 0.05 determined the statistical significance.

## Results

### Study selection process

The study selection process is summarized in Fig. 1. We identified 8382 studies from the different searched databases and excluded 6430 studies for duplication. The remaining 1952 studies were first screened by reading the title and abstract. All studies reporting the clinical characteristics (i.e. prevalence of AH, severe AH, DM, pre-DM, DL, OB, MS and CVE) of NFAI patients and of matched subjects without adrenal adenomas used as controls were evaluated for inclusion (*n* = 3). We excluded 1915 as they were meta-analysis articles (*n* = 35), case reports (*n* = 1181), review articles (*n* = 347), editorial or letters (*n* = 72) or because they were not relevant for the aims of the present meta-analysis (*n* = 280). Among the remaining 37 studies, 18 were excluded due to insufficient data since the prevalence of the outcomes was not reported [45–61], as summarized in Table 1. The inter-rate reliability between the two authors in the selection process was strong (κ = 0.86).

**Table 1:** Summary of the main characteristics of the excluded studies

Author	Country	Sample (*n*)	Reasons for Exclusion
Akkan, 2017 (ref #45)	Turkey	70	No data on outcomes prevalence and incidence in patients and control subjects
Androulakis, 2014 (ref #46)	Greece	92	Patients with diabetes and hypertension were excluded. No data on the prevalence or incidence of other outcomes
Cansu, 2017 (ref #47)	Turkey	70	Patients with diabetes, hypertension, dyslipidaemia and cardiovascular events were excluded
Dagdemir, 2023 (ref #48)	Turkey	150	No data on outcomes prevalence and incidence in patients and control subjects
Ermetici, 2008 (ref #50)	Italy	39	Controls with diabetes, hypertension and dyslipidaemia were excluded
Evran, 2016 (ref #51)	Turkey	109	Patients with diabetes, hypertension, dyslipidaemia and cardiovascular events were excluded
Imga, 2016 (ref #52)	Turkey	86	Patients with diabetes, hypertension, dyslipidaemia and cardiovascular events were excluded
Karakose, 2015 (ref #53)	Turkey	101	No data on outcomes prevalence and incidence in patients and control subjects
Kizilgul, 2017 (ref #54)	Turkey	68	No data on outcomes prevalence and incidence in control subjects
Kjellbom, 2023 (ref #55)	Sweden	4616	No data on outcomes prevalence and incidence in patients and control subjects
Li, 2021 (ref #49)	USA	1064	No data on outcomes prevalence and incidence in patients and control subjects
Marina, 2017 (ref #56)	Serbia	57	No data on outcomes prevalence and incidence in patients and control subjects
Peppa, 2010 (ref #57)	Greece	66	Patients with diabetes, hypertension, dyslipidaemia and cardiovascular events were excluded
Yener, 2009a (ref #58)	Turkey	83	No data on outcomes prevalence and incidence in patients and control subjects
Yener, 2009b (ref #59)	Turkey	82	No data on outcomes prevalence and incidence in patients and control subjects. Diabetic subjects were excluded
Yener, 2011 (ref #60)	Turkey	62	No data on outcomes prevalence and incidence in patients and control subjects
Yener, 2012 (ref #61)	Turkey	68	No data on outcomes prevalence and incidence in control subjects. Patients with diabetes and cardiovascular events were excluded

### Studies characteristics

Table 2 reports the characteristics of the 19 studies that were used in the meta-analysis [13–31]. All the 19 included studies showed cross-sectional data from 4716 subjects, mean age 57.6 ± 4.6). The outcomes incidence was given in only one study [24]. Data collection was prospective in 4 studies, retrospective in 6 studies, whereas it was not reported in 9 studies. In all studies but one [14] it was specified that the presence of other endocrine causes of AH (in particular pheochromocytoma and primary hyperaldosteronism) have been excluded.

**Table 2:** Summary of characteristics and quality evaluation by Newcastle Ottawa Scale score (NOSs) of the studies included in the metanalysis

Author and Reference	Country	Period	Sample Size (*n*)	Age (yrs)	Males (%)	AH	Severe AH	MS	DL	C-DM	CVE	NOS (0–9)
Akkus (2021)	Turkey		104	53.1	43.1	x				x		6
Anderwald (2013)	Austria	2000–2011	170	55.1	32.9					x		6
Araujo-Castro (2022)	Spain	2019–2020	48	66.5	27	x			x	x	x	6
Arduc (2014)	Turkey	2003–2010	265	50.1	22.4	x		x	x	x		6
Arruda (2018)	Brasil	2015–2016	80	56.3	25	x	x					7
Delibasi (2015)	Turkey		75	53.7	29.5	x						6
Dogra (2023)	USA	2019–2022	252	59.8	31.5	x				x	x	7
Emral (2019)	Turkey		139	54.4	35.8	x		x	x	x		7
Erbil (2009)	Turkey	2006–2008	70	49.1	9	x		x		x		8
Karatas (2023)	Turkey	2018–2020	202	53.6	33	x		x	x	x		7
Kim (2020)	Korea	2003–2012	616	55.7	73.3	x			x	x	x	8
Lopez (2016)	USA		1479	59.1	27.9	x			x	x	x	8
Moraes (2019)	Brasil	2016–2018	85	57.4	26.9	x		x	x			7
Rebelo (2023)	Brasil	2019–2021	153	61.8	22.8	x	x	x	x	x		7
Reimondo (2020)	Italy	2017–2018	577	63.3	54.9	x			x	x	x	6
Ribeiro-Cavalari (2018)	Brasil	2015–2017	164	58.7	21.2	x	x	x	x	x		6
Sokmen (2018)	Turkey	2014–2015	76	51.2	13.4	x				x		5
Szychlińska (2023)	Poland	2020	92	57.8	33.5	x		x	x			4
Tuna (2014)	Turkey	2013	69	50.5	27.4	x						5

AH. arterial hypertension; MS. metabolic syndrome; DL. dyslipidemia; C-DM. diabetes mellitus and/or prediabetes; CVE. cardiovascular events. NOS. Newcastle-Ottawa Scale

From these studies the AH, severe AH, DM, preDM, DM, DM and/or preDM (composite DM, C-DM), DL, OB, MS and CVE prevalence was obtained. Among these studies, the association between the prevalence of AH, severe AH, DM, pre-DM, composite DM, DL, OB, MS and CVE was reported in 18, 3, 7, 2, 5, 4, 11, 8 and 5 studies, respectively. Given the low number of studies including data on pre-DM (*n* = 2), we decided to evaluate the association between the presence of NFAI and the prevalence of DM and C-DM only. The prevalence of obesity was reported in only 4 [15, 16, 19, 20] and, thus, we decided not to include obesity as possible additional outcome in the study.

The geographic areas of the included studies were Europe (*n* = 12), East Asia (*n* = 1), South America (*n* = 4), North America (*n* = 2). The quality of included studies varied (Newcastle-Ottawa Scale between 6 and 8).

The measured outcomes sample sizes, and number of cases meeting outcomes in patients with NFAI and control subjects, evaluated in the 19 included studies, are summarized in Table 3.

**Table 3:** Number of included studies and sample size considered for each specific outcome

Outcome	Included Studies *n*	Total Subjects *n*	Cases *n*	Controls *n*	Cases meeting the outcome *n* (%)	Controls meeting the outcome *n* (%)
**AH**	18	4546	1416	3130	698 (49.3)	1295 (41.4)
**DM**	7	1743	481	1262	111 (23.1)	176 (14.0)
**C-DM**	14	4315	1361	2954	481 (35.3)	546 (18.5)
**DL**	11	3820	1025	2795	527 (51.4)	1052 (37.6)
**MS**	8	1170	620	550	342 (55.2)	175 (31.8)
**CVE**	5	2972	603	2369	75 (12.4)	335 (14.1)

AH: arterial hypertension. DM: diabetes mellitus. C-DM (composite DM): diabetes mellitus and/or impaired fasting glucose and/or glucose intolerance. DL: dyslipidemia. MS: metabolic syndrome (for definitions see ref #38–41). CVE: cardiovascular events (myocardial infarction, stroke, transient ischemic attack, angina pectoris, pulmonary embolism, intracerebral hemorrhage, and peripheral artery disease)

Cases: Patients with non-functioning adrenal incidentaloma (NFAI). Controls: subjects without NFAI. Cases meeting the outcome: number (and percentage of patients in parenthesis) of NFAI patients with AH, DM, C-DM, DL, MS and CVE. Controls meeting the outcome: number (and percentage of controls in parenthesis) of subjects without NFAI with AH, DM, C-DM, DL, MS and CVE

Seven studies included some of the predefined outcomes among the exclusion criteria: the presence of DM, CVE, DM and CVE was among the exclusion criteria in 4 [14, 17, 18, 30], 5 [13, 18, 20, 29, 30] and 3 [18, 20, 30] studies respectively. These studies were anyway included in the meta-analysis, using their data regarding the available outcomes.

Control groups were age-, sex-matched in 2 studies [23, 29], age-, sex-, BMI-matched in 5 studies [14, 16, 18, 21, 22], age-, sex-, ethnicity-matched in 1 study [24] and age-, sex-, BMI, ethnicity-matched in 1 study [30]. Control individuals were not matched with patients with NFAI in 7 studies [17, 19, 20, 25–28], and in 3 studies the matching procedure was not specified [13, 15, 31].

### Association between the NFAI presence and the prevalence of the investigated outcomes

**Fig. 2:**
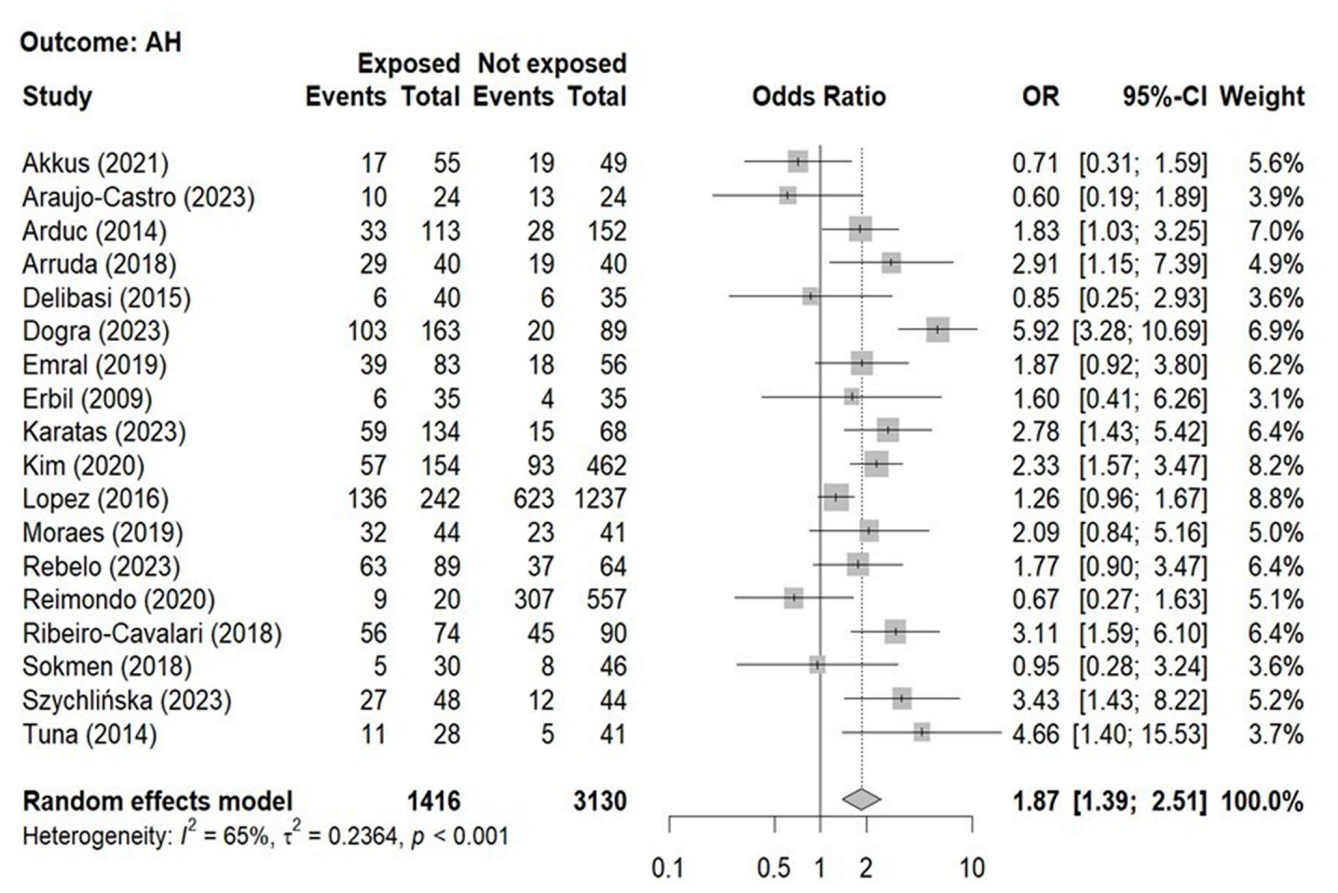
Forest-plot illustrating the association between the prevalence of arterial hypertension and the presence non-functioning adrenal tumors. Footnotes: The DerSimonian and Laird method for calculating the summary association estimates and their 95% Confidence Intervals (95%CI) has been used

The overall prevalence of AH in patients with NFAI (49.3%) as compared with controls (41.4%) derives from 18 studies [13, 15–31] including 4546 subjects (1416 patients with NFAI, 3130 controls, Table 3). The forest plot illustrating the association between the prevalence of AH in patients with NFAI as compared to controls is shown in Fig. 2. Patients with NFAI showed a 1.9-fold increased prevalence of AH as compared to control subjects (Table 4). Three studies included in this meta-analysis reported the prevalence of severe AH in patients with NFAI and control individuals [17, 26, 28] and showed a very highly increased risk of severe AH in patients with NFAI than in controls (OR 5.02, 95%CI 1.91–13.23).

**Table 4:** Odds ratio for the associations between the presence of a non-functioning adrenal incidentaloma and the presence of arterial hypertension, diabetes mellitus, composite diabetes mellitus, dyslipidaemia, metabolic syndrome and cardiovascular events

	OR	95%CI	I2	Studies (*n*)
**AH**	1.87	1.39–2.51	65%	18
**Severe AH**	5.02	1.91–13.23	65%	3
**DM**	1.57	0.70–3.54	70%	7
**C-DM**	2.04	1.70–2.45	50%	14
**DL**	1.23	0.95–1.58	40%	11
**MS**	2.89	1.93–4.32	52%	8
**CVE**	1.22	0.71–2.08	36%	5

OR: odds ratio, 95%CI: 95% confidence Interval; I2: grade of heterogeneity

AH: arterial hypertension. Severe AH: resistant AH or AH treated with ≥ 3 drugs. DM: diabetes mellitus. C-DM (composite DM): diabetes mellitus and impaired fasting glucose and/or glucose intolerance. DL: dyslipidemia. MS: metabolic syndrome (for definitions see ref #38–41). CVE: cardiovascular events (myocardial infarction, stroke, transient ischemic attack, angina pectoris, pulmonary embolism, intracerebral hemorrhage, and peripheral artery disease)

**Fig. 3:**
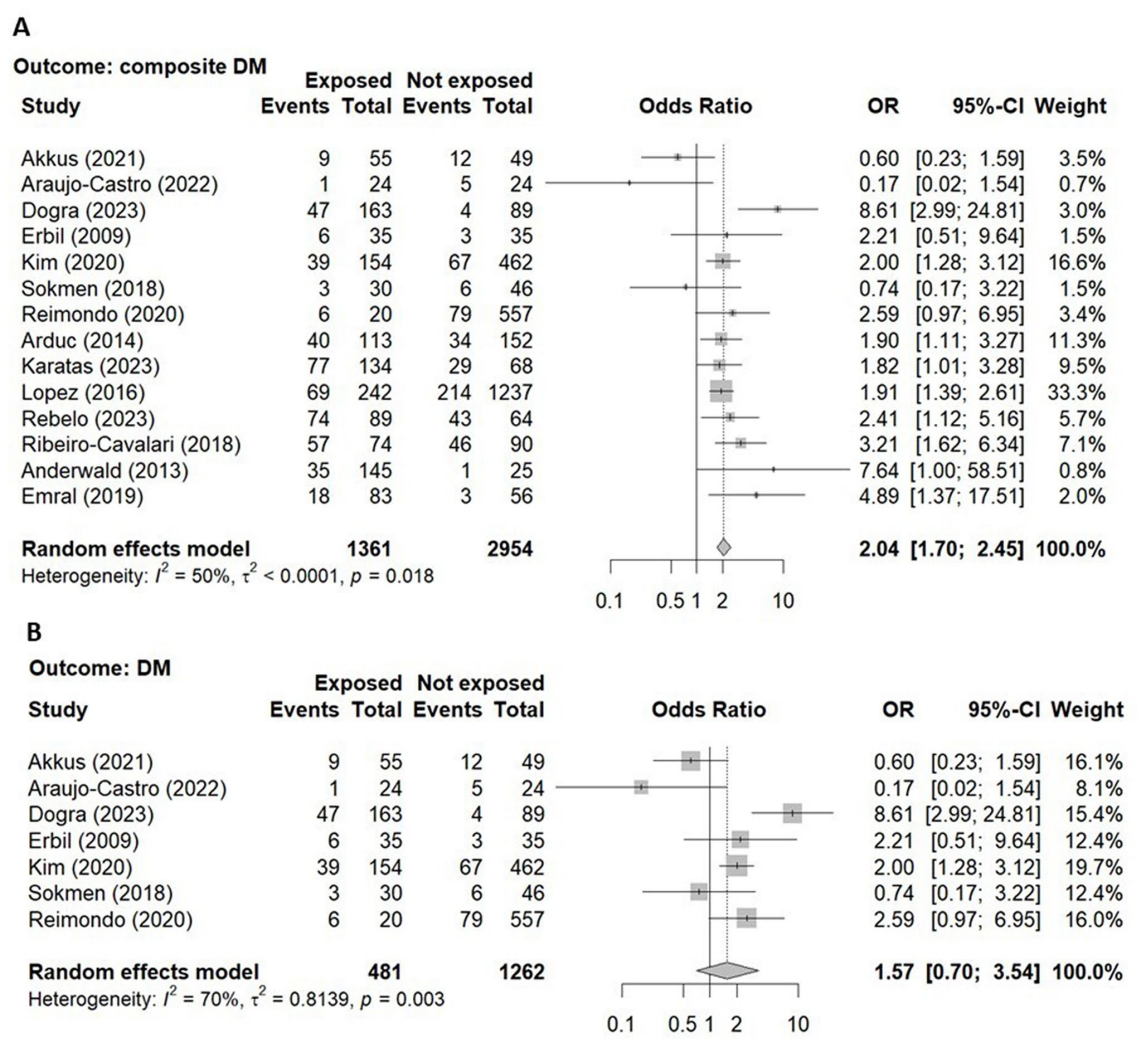
Forest-plots illustrating the association between the prevalence of diabetes mellitus **(panel A)** and composite diabetes mellitus **(panel B)** and the presence non-functioning adrenal tumors. Footnotes: The DerSimonian and Laird method for calculating the summary association estimates and their 95% Confidence Intervals (95%CI) has been used. Composite diabetes mellitus: diabetes mellitus and/or impaired fasting glucose and/or glucose intolerance

The overall prevalence of C-DM in patients with NFAI (35.3%) as compared with controls (18.5%) derives from 14 studies [13–16, 19–24, 26–29] including 4315 subjects (1361 NFAI patients, 2954 controls, Table 3). The forest plot illustrating the association between the prevalence of C-DM in patients with NFAI and controls is shown in Fig. 3a. Patients with NFAI showed a 2-fold increased prevalence of C-DM than control subjects (Table 4). Seven studies [13, 15, 19, 21, 23, 27, 29] reported the prevalence of DM in patients with NFAI (23.1%) and control subjects (18.5%, Table 3). The association between the presence of DM and the presence of NFAI was not statistically significant (Fig. 3b; Table 4).

**Fig. 4:**
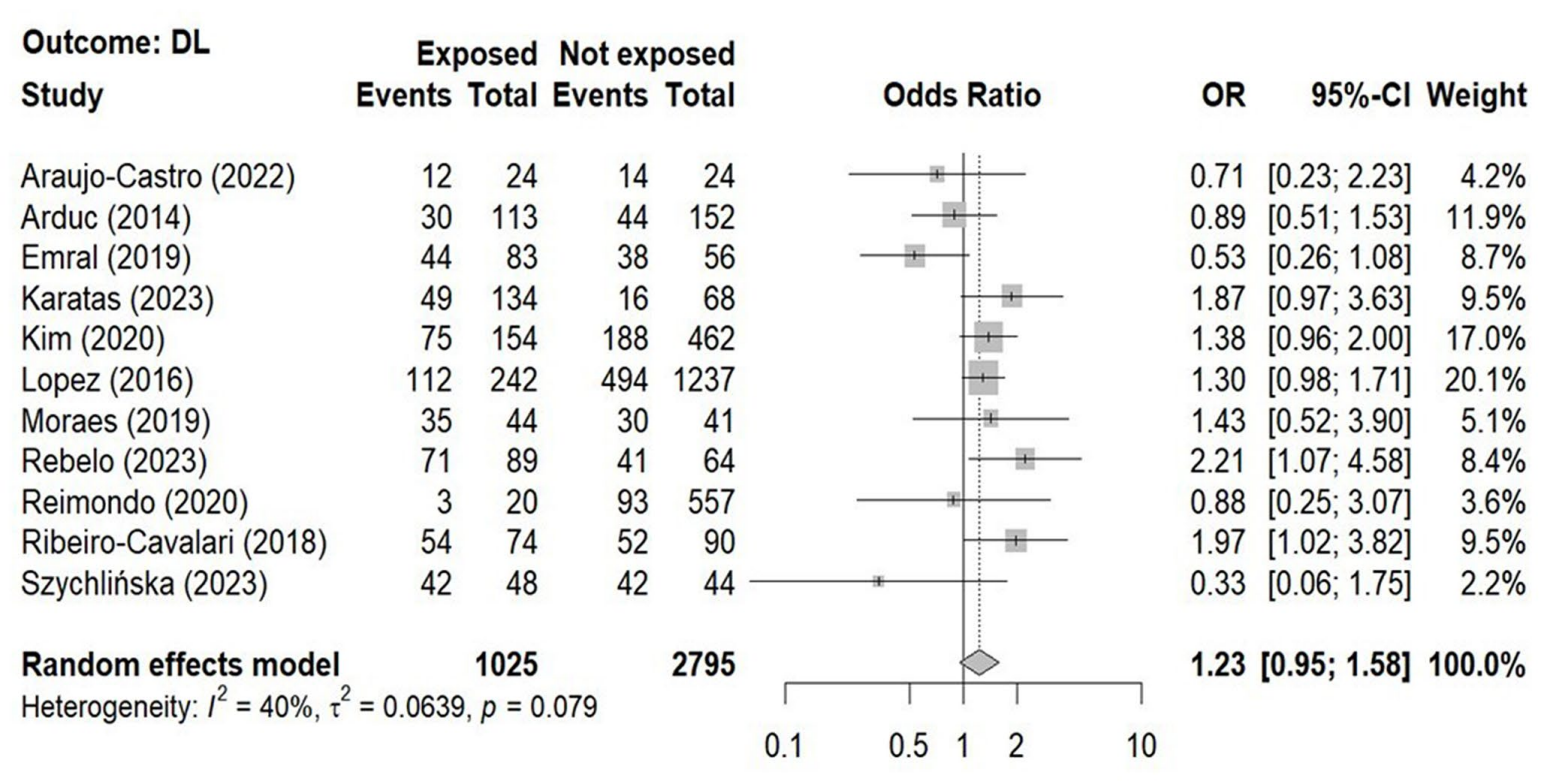
Forest-plot illustrating the association between the prevalence of dyslipidemia and the presence non-functioning adrenal tumors. Footnotes: The DerSimonian and Laird method for calculating the summary association estimates and their 95% Confidence Intervals (95%CI) has been used. The presence of dyslipidemia was based on the criteria released by Expert Panel on Detection, Evaluation, And Treatment of High Blood Cholesterol In Adults [42]

The overall prevalence of DL in patients with NFAI (51.4%, 3820 subjects) and controls (37.6%, 1052 subjects, Table 3) was calculated from 11 studies [15, 16, 20, 22–28, 30]. The relative forest plot shows that the DL prevalence was not statistically different between patients with NFAI and control subjects (Fig. 4; Table 4).

**Fig. 5:**
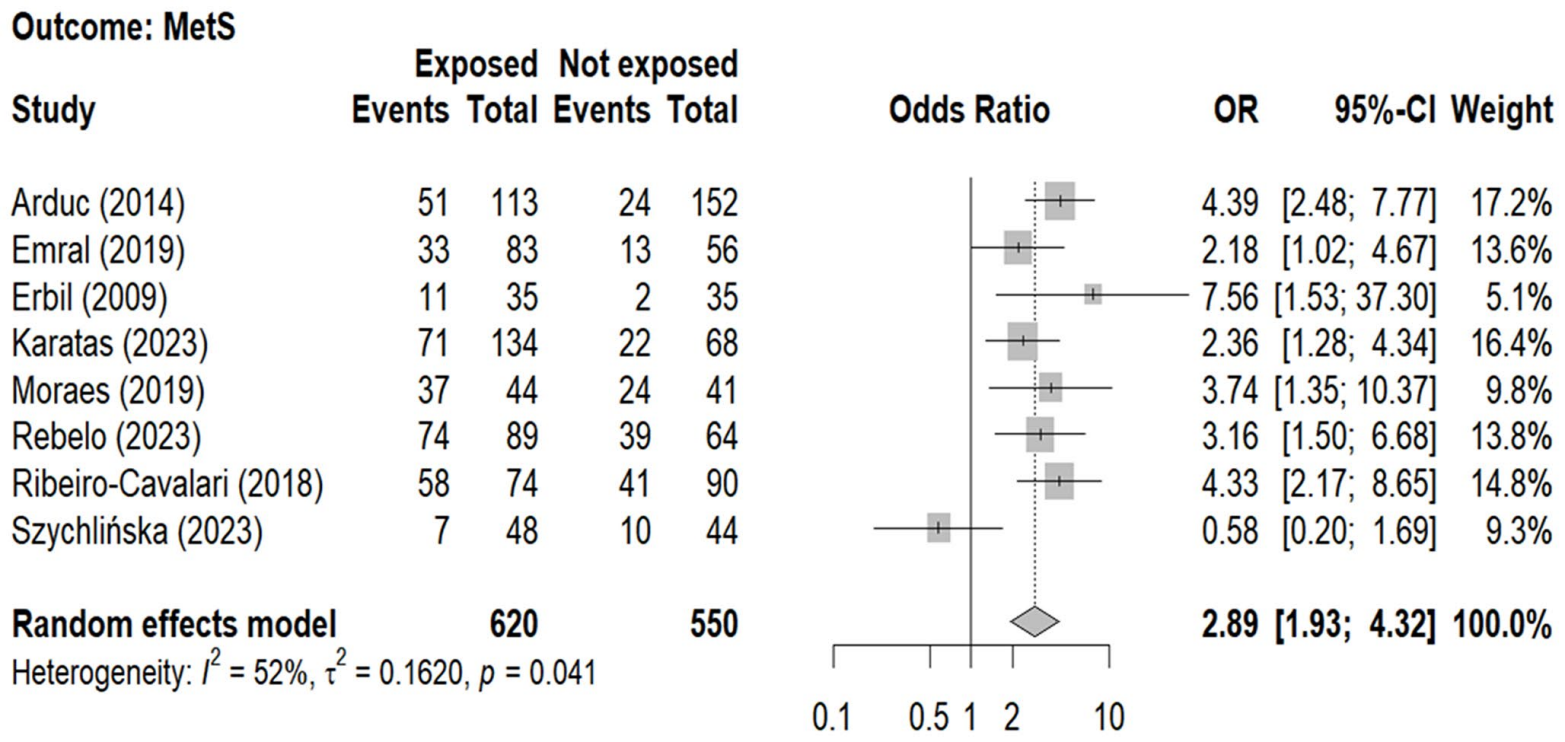
Forest-plot illustrating the association between the prevalence of metabolic syndrome and and the presence non-functioning adrenal tumors. Footnotes: The DerSimonian and Laird method for calculating the summary association estimates and their 95% Confidence Intervals (95%CI) has been used. The metabolic syndrome diagnosis was based on the criteria by the American Diabetes Association 2023 [41]

The overall prevalence of MS was higher in patients with NFAI (55.2%) than in controls (31.8%). The forest plot (Fig. 5) included 8 studies [16, 20–22, 25, 26, 28, 30] (1170 subjects, 620 NFAI patients, 550 controls) and showed that patients with NFAI were about 3-fold times more frequently affected by MS than controls (Table 4).

**Fig. 6:**
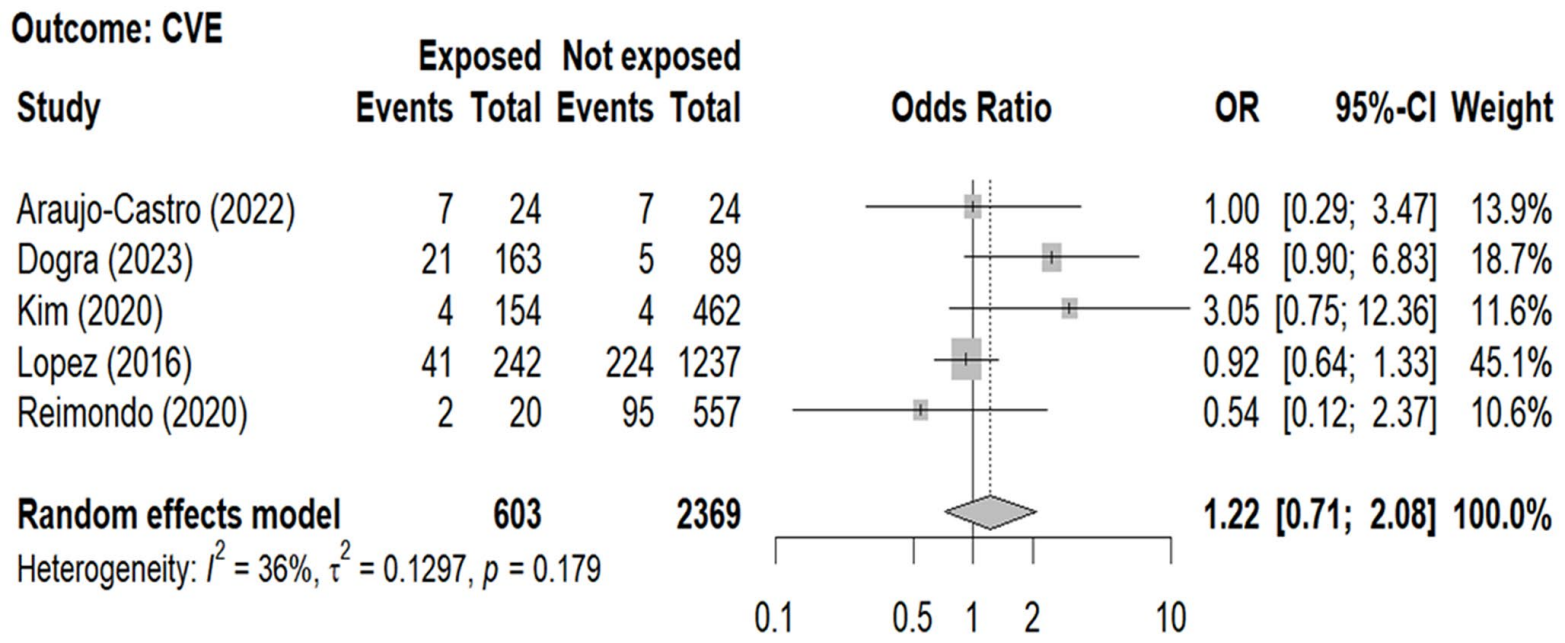
Forest-plot illustrating the association between the prevalence of cardiovascular events and and the presence non-functioning adrenal tumors. Footnotes: The DerSimonian and Laird method for calculating the summary association estimates and their 95% Confidence Intervals (95%CI) has been used. Cardiovascular events: myocardial infarction, stroke, transient ischemic attack, angina pectoris, pulmonary embolism, intracerebral hemorrhage, and peripheral artery disease

The overall prevalence of CVE was calculated from 5 studies [15, 19, 23, 24, 27] and it was found to be similar in patients with NFAI and controls (603 subjects, 12.4% and 2369 subjects, 14.1%, respectively) as shown in Fig. 6; Table 4.

For all the outcomes, meta-regression analysis did not show any association between the covariates (age, gender and BMI) and ORs with the only exception of gender which affected the OR between NFAI and MetS: as the proportion of males (in the NFAI + or in the NFAI-) increases the OR tends to decrease (*p* = 0.032 and *p* = 0.007, respectively), as shown in supplementary Table 1.

The analysis of funnel plots did not reveal any clear asymmetry, suggesting the absence of publication bias possibly influencing either AH, or or C-DM or MS (supplementary Figure). Likewise, the influence analysis did not show an impact of each study-specific association estimate on the pooled OR (supplementary Table 2).

## Discussion

The present systematic review and meta-analysis shows that AH, C-DM, and MS, are significantly more frequent in patients with NFAI than in control individuals without adrenal tumours, suggesting that a certain degree of cortisol hypersecretion may be present in some patients with NFAI.

The idea that these so called “not-functioning” adrenal tumors may, in fact, secrete a certain amount of cortisol arose from sparse data showing that patients with NFAI may have a worse cardiometabolic profile, including a higher risk of AH [16, 17, 19, 22, 23, 28, 30, 31], DM [19, 23], DL [24, 28], MS [16, 20–22, 25, 26, 28] and cardiovascular alterations [46] than control individuals. However, the same studies were not consistent in demonstrating a statistically significant difference in the prevalence of all these comorbidities between patients with NFAI and control individuals. A recent meta-analysis suggested that patients with NFAI presented higher odds of DM than healthy controls [62], but a comprehensive meta-analysis focused on clarifying whether patients with NFAI are at higher risk of being affected by all cardiometabolic comorbidities was not available in the literature so far.

The present finding of the association between the presence of NFAI and a worse cardiometabolic profile is in keeping with a large (17726 cases and 124366 controls) retrospective register-based case-control study by Patrova and coauthors suggesting that the mortality risk is 1.4 increased in patients with NFAI as compared to controls in particular for cardiovascular disease and malignancy [32]. As pointed out by other authors [63], however, in the study by Patrova and coauthors the lack of a hormonal evaluation or of radiological report may have biased the results since some individuals may have received a NFAI diagnosis despite potentially having a slight hypercortisolism. In addition, even though the authors excluded subjects with a cancer diagnosis within the first three months from NFAI diagnosis, this could not have been enough to exclude that patients with cancer-related symptoms had underwent a radiological imaging more frequently [32]. Interestingly, previous data suggested that the mortality risk in patients with NFAI was similar to that described in subjects with MACS [35]. Moreover, the theory that some patients with NFAI may have a certain degree of hypercortisolism is even supported by the finding that adrenalectomy may improve blood pressure and glucometabolic control in some patients with NFAI [62] and that these patients may be at risk of post-surgical hypocortisolism after the removal of the adrenal mass [34].

From a clinical point of view, the present estimate of the increased risk of AH, DM and/or preDM and MS in AI patients without apparent hypercortisolism is of importance to shed light on some patients who, based on the available guidelines [2], may be considered not to be followed up over time. Importantly, three studies included in this meta-analysis reported the prevalence of severe AH (i.e. resistant AH or AH treated with ≥ 3 drugs) in patients with NFAI and control individuals [17, 26, 28] and showed a 5-fold increased risk of having severe AH in patients with NFAI than in controls. This finding further confirms that, as MACS patients, even patients with NFAI deserve to be controlled as far as the comorbidities of a possible cortisol hypersecretion is concerned.

At variance with the meta-analysis by Athanasouli and coauthors [62], we did not find an increased prevalence of DM alone but only of DM and/or Pre-DM in patients with NFAI. This is probably explained by the fact that Athanasouli and coauthors included in their meta-analysis two studies reporting data on the prevalence of DM and Pre-DM [16, 28] rather than of DM only, and that we also added two studies [13, 19] that were not included in the Athanasouli meta-analysis. Overall, it is possible to hypothesize that the lack of a statistically significant difference in the DM prevalence between patients with NFAI and control individuals in the present analysis could be related to the low number of available data (111 cases meeting the outcome). Similarly, the low number of studies reporting CVE (*n* = 5) and the low number of cases meeting the outcome (*n* = 75) may have contributed to the lack of a statistically significant difference in CVE between patients with NFAI and control subjects. In general, given the multifactorial and complex pathophysiology of CVE, it is conceivable that large samples of well-defined patients with NFAI would be needed for clarifying whether these patients have an increased prevalence of CVE.

The reason for patients with NFAI to be at higher risk of cardiometabolic comorbidities could be due to the low sensitivity of the criteria used for diagnosing MACS (i.e. F-1mgDST > 1.8 µg/dL, 50 nmol/L) [2]. Indeed, it is likely that in patients with AI, a continuum from inactive tumors to MACS exists and, therefore, the risk of being affected by cardiometabolic comorbidities increases with the low-grade increase of cortisol even within ranges, that we still consider to be normal [24]. This idea is further supported by the recent finding that, among patients with NFAI, subjects with F-1mgDST levels between 1.2 µg/dL (33 nmoL/L) and 1.79 µg/dL (49 nmol/L) seem to have a higher prevalence of AH and DM and a worse cardiometabolic profile than patients with AI and F-1mgDST levels < 1.2 µg/dL (33 nmoL/L), even though the F-1mgDST levels set at 1.2 µg/dL (33 nmoL/L) had a low diagnostic accuracy [64]. Likewise, previous data suggested that in patients with AI, who underwent surgery, only a F-1mgDST as low as < 1.2 µg/dL (33 nmol/L) ruled out with 100% sensitivity the occurrence of a post-surgical hypocortisolism. Of note in that study about 29% (9/31) of patients with NFAI and F-1mgDST between 1.2 and 1.8 µg/dL (33–50 nmol/L), who were operated on for the size of the adenoma, has a post-surgical hypocortisolism, suggesting that a F-1mgDST < 1.2 µg/dL (33.1 nmol/L) rules could be used to exclude hypercortisolism in AI patient [34]. Moreover, it has been reported that in patients with AI the best accuracy for predicting cardiovascular risk and insulin resistance was obtained by using a cut-off of cortisol after two days low dose dexamethasone suppression test set at 1.4 µg/dL (39 nmol/L) and 1.1 µg/dL (30 nmol/L) respectively [46]. Finally, previous data showed that the F-1mgDST cut-off with the best compromise between sensitivity and specificity for predicting CVE in patients with AI was found to be as low as 1.5 µg/dL (41 nmol/L) [65]. Thus, based on the present findings and past data it could be possible to hypothesize that among the so called “non-functioning” adrenal tumours, some patients display a certain degree of cortisol hypersecretion. If these data would be confirmed, the F-1mgDST cut-off for correctly defining patients with NFAI should be lowered to below 1.5 µg/dL (39 nmol/L) till 1.2 µg/dL (33.1 nmol/L). However, reducing the cut-off of F-1mgDST for diagnosing MACS in patients with AI would increase the rate of false positive results. Thus, until more advanced techniques, such as mass spectrometry, the measurement of dexamethasone in blood and the use of reliable markers of autonomous cortisol secretion, such as adrenocorticotroph hormone and dehydroepiandrosterone hormone levels, are introduced, the practical usefulness of lowering the F-1mgDST cut-off will need to be better elucidated.

This meta-analysis has some intrinsic limitations. Firstly, to date, no interventional studies exist on the effect of surgery in NFAI and, thus, we could include only observational studies. Since these latter cannot prove causality, interventional studies are of key importance. At this regard, although a previous metanalysis suggested that AH and DM may be ameliorated by surgery in some patients with AI even in the absence of subclinical hypercortisolism [33], it must be observed that the criteria used for defining the absence of subclinical hypercortisolism in the studies included were different from those currently used for defining the absence of MACS. Therefore, the beneficial effect of surgery on AH and DM suggested by previous data in patients without subclinical hypercortisolism has still to be demonstrated in patients without MACS. Secondly, the present meta-analysis could analyse only cross-sectional studies, as too few longitudinal studies were available. However, all the included studies were of good quality according to the modified Newcastle-Ottawa scale (score of 6 or higher). Moreover, at least for DM, the study by Lopez and coauthors showed that patients with NFAI (*n* = 242) were at higher risk for incident C-DM when compared with patients without adrenal tumour [24]. On the other hand, a smaller study (*n* = 115 subjects) by Kim and colleagues failed to find differences in incident AH and DM in patients with NFAI as compared to control individuals [23]. Thirdly, the 5 studies [15, 19, 23, 24, 27] reporting the prevalence of CVE in patients with NFAI and in controls, did not specifically report the prevalence of the different types of cardiovascular events, which could have been even more informative. Moreover, in some studies [13, 14, 17, 18, 20, 29, 30] patients affected by T2D and/or CVE have been excluded, thus potentially reducing the number of subjects at risk of comorbidities. However, the influence analysis did not show an impact of each study-specific association estimate on the pooled OR. A fourth limitation of the present meta-analysis is that only two prospective studies assessed the mortality risk in patients with NFAI as compared to patients without adrenal tumours. Kjellbom and colleagues in a retrospective study on 1154 patients and 3462 matched controls with a median follow-up of 6.6 years did not show a statistically significant difference in mortality between patients with NFAI and their controls. At variance, in a larger case-control registry study on 17726 cases and 124366 controls, overall mortality was higher in patients with NFAI than in controls (adjusted hazard ratio, 1.21; 95%CI, 1.16–1.26, median follow-up of 6.2 years).

Notwithstanding these study limitations, the present data are important since, for the first time, they systematically show that patients with NFAI have higher prevalence of AH, C-DM and MS than control subjects without NFAI. These findings suggest that “non-functioning” adrenal tumours are probably secreting some amounts of excess cortisol and, therefore, the term “non-functioning” should not be used at least for a subset of currently defined patients with NFAI. If these data will be confirmed, patients with NFAI without comorbidities without comorbidities at the initial evaluation will need to be followed up over time. On the basis of the not-negligible risk of MACS development over time reported in studies with an adequate follow-up and since the F-1mgDST is an easy-to-perform and low-cost test, we believe that in patients with NFAI a clinical evaluation and the F-1mgDST should be performed every year and every two years, respectively, for at least five years [66].

Further longitudinal studies should be designed to assess: (i) the risk of incident cardiovascular comorbidities and mortality in patients NFAI; (ii) whether the cut-off of F-1mgDST must be lowered and until what point in order to increase the diagnostic sensitivity; (iii) whether other markers of glucocorticoid hypersecretion may be helpful for individuating patients with AI at higher risk of cortisol-related comorbidities.

## Electronic supplementary material

Below is the link to the electronic supplementary material.

Supplementary Material 1: Fig. 1. Funnel Plots evaluating the possible publication bias on the association between either arterial hypertension, or composite diabetes or metabolic syndrome and the presence non-functioning adrenal tumors. Footnotes: Composite diabetes mellitus: diabetes mellitus and/or impaired fasting glucose and/or glucose intolerance. Metabolic syndrome was reported based on the criteria American Diabetes Association 2023 [41].

Supplementary Material 2: Table 1. Meta-regression analysis to assess the impact of several covariates (including age, gender and body mass index) on the pooled odds ratio.

Supplementary Material 3: Table 2. Influence analysis to assess of the impact of each study-specific association estimate on the pooled odds ratio.
