# Supplementary figures and images for: Cardiometabolic comorbidities and cardiovascular events in “non-functioning” adrenal incidentalomas: a systematic review and meta-analysis
Source: J Endocrinol Invest. 2024 Sep 30;47(12):2929–42. doi: 10.1007/s40618-024-02440-0 (PMC11549128; doi:10.1007/s40618-024-02440-0)

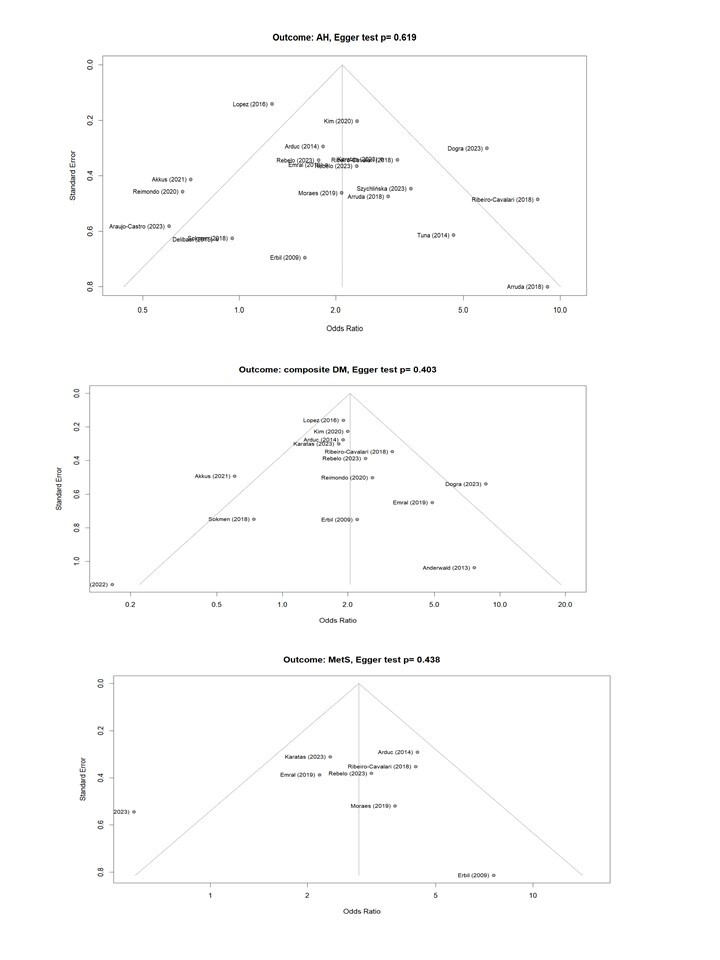

Supplement: Supplementary file 1 — Supplementary Material 1: Fig. 1. Funnel Plots evaluating the possible publication bias on the association between either arterial hypertension, or composite diabetes or metabolic syndrome and the presence non-functioning adrenal tumors. Footnotes: Composite diabetes mellitus: diabetes mellitus and/or impaired fasting glucose and/or glucose intolerance. Metabolic syndrome was reported based on the criteria American Diabetes Association 2023 [41]. [file 40618_2024_2440_MOESM1_ESM.jpg]
