# Supplementary material for: Cardiometabolic comorbidities and cardiovascular events in “non-functioning” adrenal incidentalomas: a systematic review and meta-analysis
Source: J Endocrinol Invest. 2024 Sep 30;47(12):2929–42. doi: 10.1007/s40618-024-02440-0 (PMC11549128; doi:10.1007/s40618-024-02440-0)

**Meta-regression**

*Only for otucomes with a significant association with NFAI*

*We studied OR as a function of:*

- *mean or median age in the NFAI+*
- *mean or median age in the NFAI-*
- *proportion of males in the NFAI+*
- *proportion of males in the NFAI-*
- *mean or median BMI in the NFAI+*
- *mean or median BMI in the NFAI-*

The only association found is between the OR of MetS and the gender: as the proportion of males (in the NFAI+ or in the NFAI-) increases, the OR tends to decrease (p= 0.032 and p= 0.007, respectively).

**Outcome AH (not severe)**

**
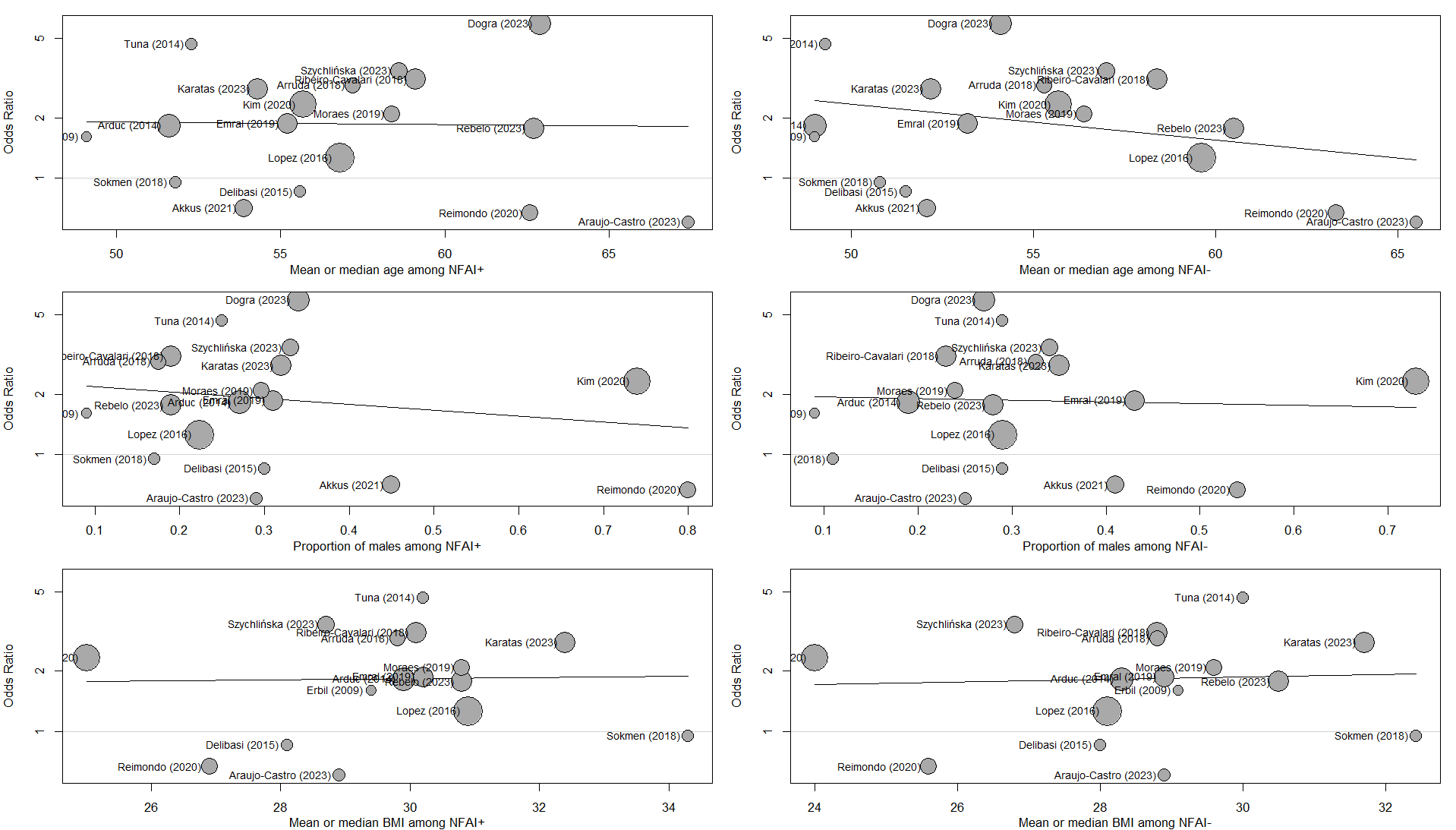
**

**AH or severe AH**

**
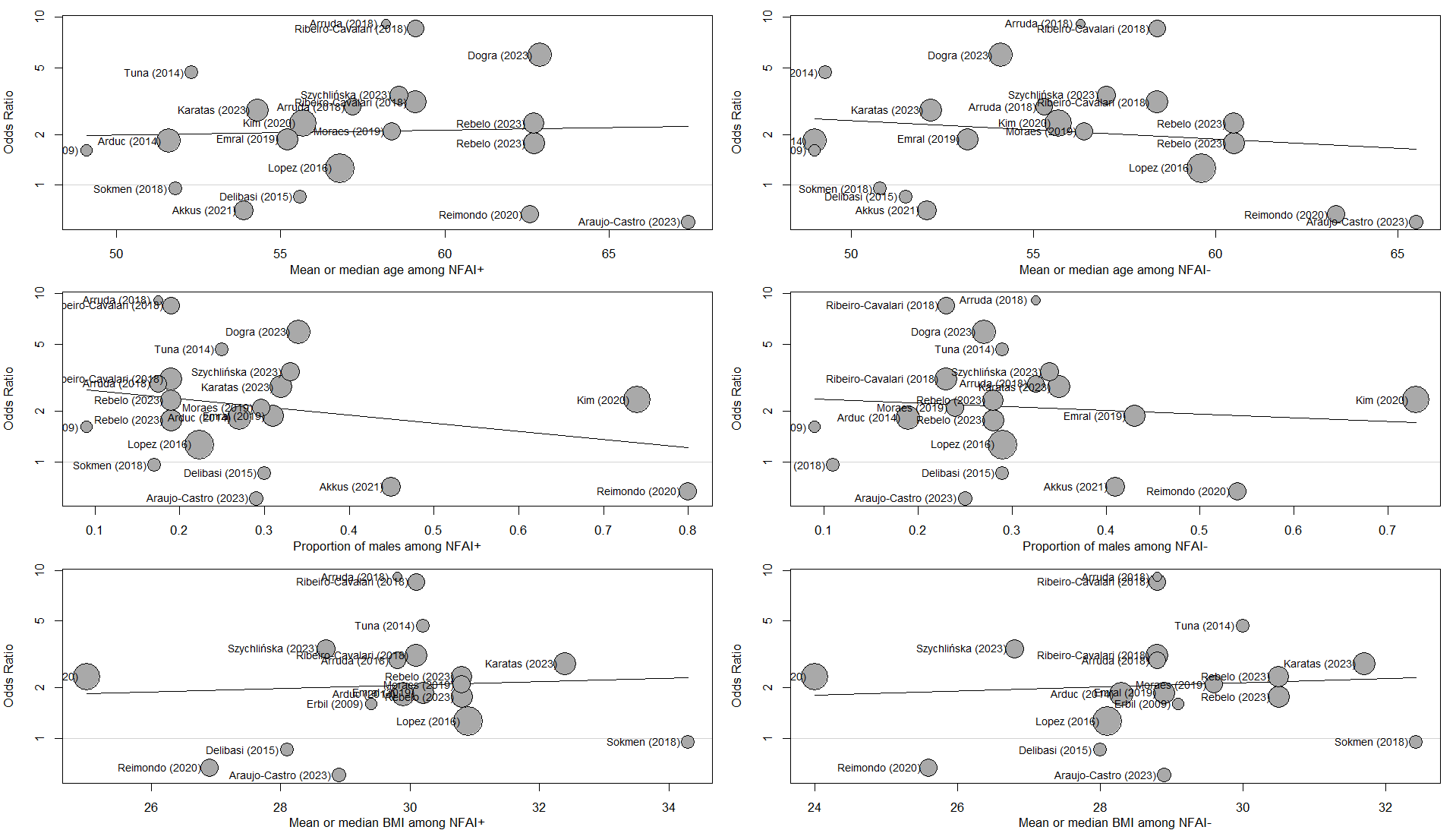
**

**MetS**


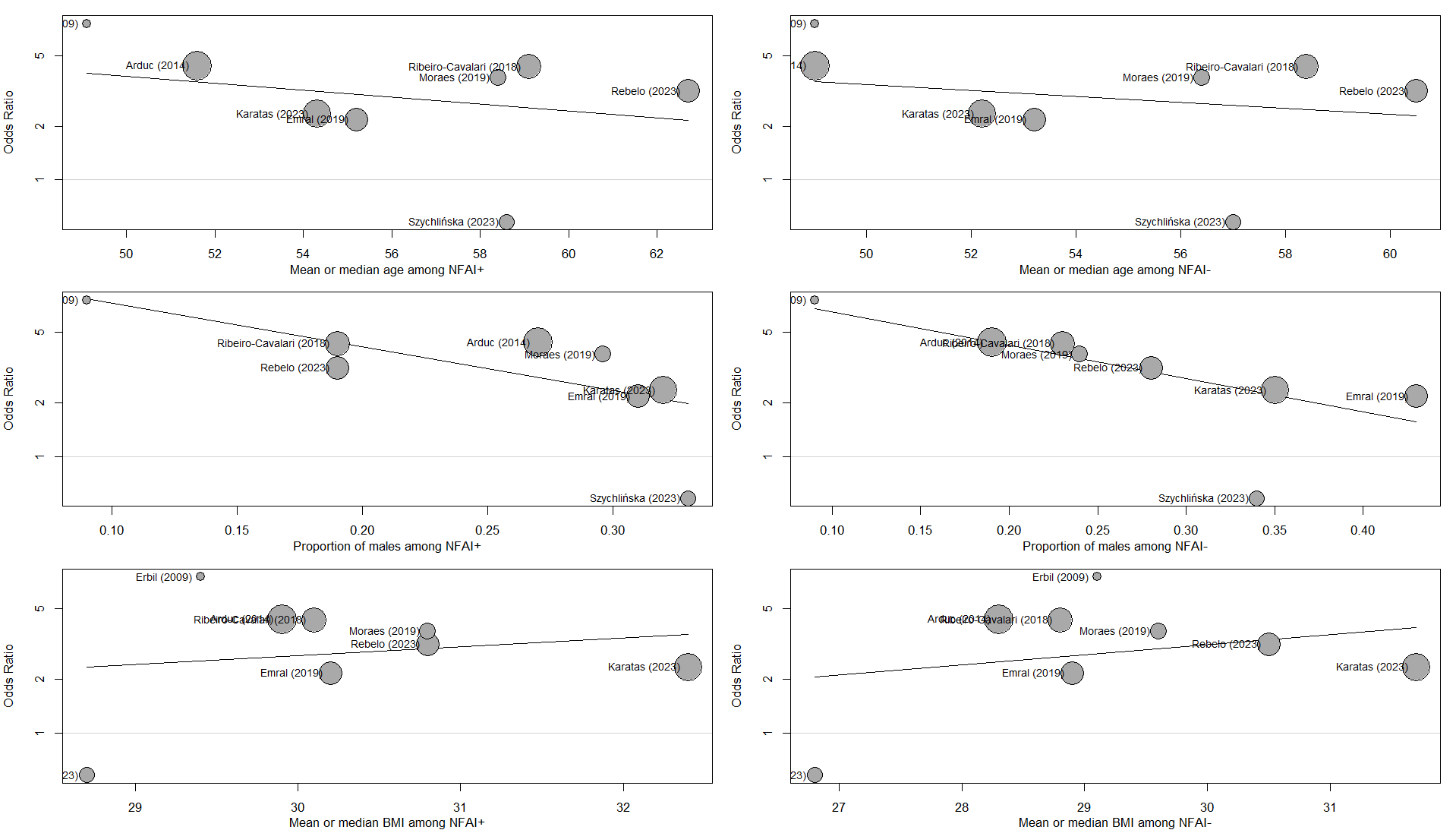


**Composite DM**


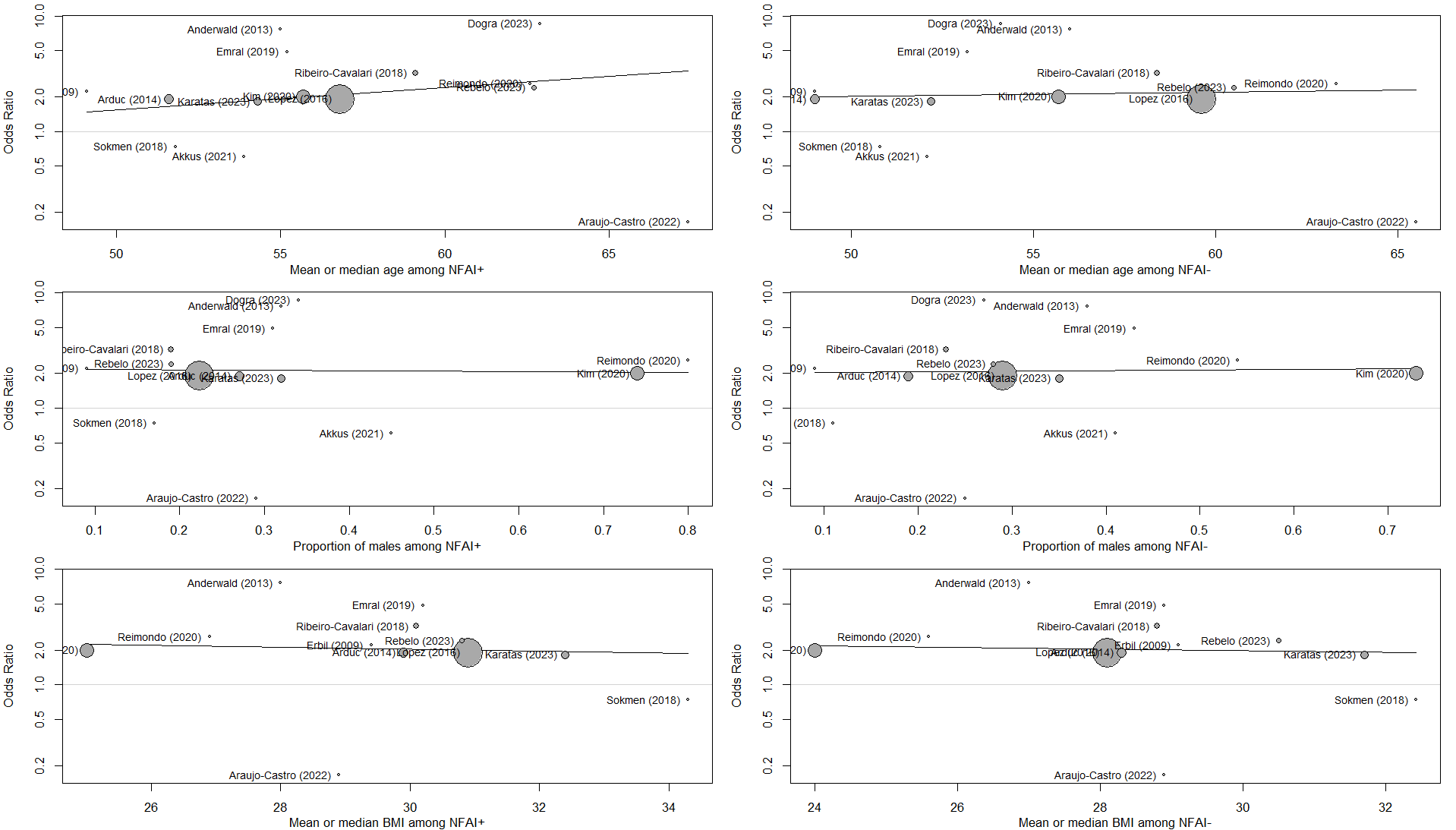

Supplement: Supplementary file 2 — Supplementary Material 2: Table 1. Meta-regression analysis to assess the impact of several covariates (including age, gender and body mass index) on the pooled odds ratio. [file 40618_2024_2440_MOESM2_ESM.docx]
