# Supplementary material for: Cardiometabolic comorbidities and cardiovascular events in “non-functioning” adrenal incidentalomas: a systematic review and meta-analysis
Source: J Endocrinol Invest. 2024 Sep 30;47(12):2929–42. doi: 10.1007/s40618-024-02440-0 (PMC11549128; doi:10.1007/s40618-024-02440-0)
Supplement: Supplementary file 3 — Supplementary Material 3: Table 2. Influence analysis to assess of the impact of each study-specific association estimate on the pooled odds ratio. [file 40618_2024_2440_MOESM3_ESM.docx]

**INFLUENCE ANALYSIS (LEAVE-ONE OUT METHOD)**

**Outcome AH (not severe)**

OR 95%-CI p-value tau^2 tau I^2

Omitting Akkus (2021) 1.9879 [1.4931; 2.6466] < 0.0001 0.1953 0.4419 63.3%

Omitting Araujo-Castro (2023) 1.9614 [1.4664; 2.6235] < 0.0001 0.2121 0.4605 64.8%

Omitting Arduc (2014) 1.8681 [1.3588; 2.5683] 0.0001 0.2684 0.5181 67.4%

Omitting Arruda (2018) 1.8253 [1.3411; 2.4844] 0.0001 0.2511 0.5011 66.7%

Omitting Delibasi (2015) 1.9258 [1.4272; 2.5987] < 0.0001 0.2347 0.4845 66.4%

Omitting Dogra (2023) 1.7307 [1.3425; 2.2311] < 0.0001 0.1220 0.3492 51.0%

Omitting Emral (2019) 1.8654 [1.3603; 2.5579] 0.0001 0.2651 0.5149 67.4%

Omitting Erbil (2009) 1.8775 [1.3836; 2.5477] < 0.0001 0.2503 0.5003 67.4%

Omitting Karatas (2023) 1.8173 [1.3303; 2.4825] 0.0002 0.2541 0.5041 66.3%

Omitting Kim (2020) 1.8298 [1.3290; 2.5193] 0.0002 0.2677 0.5173 66.2%

Omitting Lopez (2016) 1.9399 [1.4191; 2.6518] < 0.0001 0.2460 0.4960 59.6%

Omitting Moraes (2019) 1.8554 [1.3582; 2.5346] 0.0001 0.2598 0.5097 67.3%

Omitting Rebelo (2023) 1.8725 [1.3649; 2.5688] 0.0001 0.2654 0.5152 67.4%

Omitting Reimondo (2020) 1.9822 [1.4878; 2.6410] < 0.0001 0.1987 0.4458 63.7%

Omitting Ribeiro-Cavalari (2018) 1.8050 [1.3259; 2.4573] 0.0002 0.2456 0.4956 65.6%

Omitting Sokmen (2018) 1.9181 [1.4189; 2.5928] < 0.0001 0.2390 0.4888 66.6%

Omitting Szychlińska (2023) 1.8081 [1.3325; 2.4535] 0.0001 0.2422 0.4921 65.9%

Omitting Tuna (2014) 1.8068 [1.3394; 2.4374] 0.0001 0.2336 0.4833 65.7%

Pooled estimate 1.8704 [1.3926; 2.5120] < 0.0001 0.2364 0.4862 65.3%

**Severe AH**

OR 95%-CI p-value tau^2 tau I^2

Omitting Arruda (2018) 4.2781 [1.2005; 15.2461] 0.0250 0.6593 0.8119 78.1%

Omitting Rebelo (2023) 8.6861 [3.8490; 19.6023] < 0.0001 0.0000 0.0000 0.0%

Omitting Ribeiro-Cavalari (2018) 3.8324 [1.0520; 13.9613] 0.0417 0.5518 0.7428 58.8%

Pooled estimate 5.0215 [1.9063; 13.2276] 0.0011 0.4491 0.6701 64.8%

**AH or severe AH**

OR 95%-CI p-value tau^2 tau I^2

Omitting Akkus (2021) 2.2123 [1.6573; 2.9532] < 0.0001 0.2500 0.5000 66.6%

Omitting Araujo-Castro (2023) 2.1898 [1.6331; 2.9362] < 0.0001 0.2685 0.5182 67.8%

Omitting Arduc (2014) 2.1097 [1.5343; 2.9007] < 0.0001 0.3320 0.5762 69.8%

Omitting Arruda (2018) 2.0598 [1.5081; 2.8132] < 0.0001 0.3190 0.5648 69.5%

Omitting Delibasi (2015) 2.1572 [1.5948; 2.9178] < 0.0001 0.2950 0.5432 69.0%

Omitting Dogra (2023) 1.9538 [1.4785; 2.5819] < 0.0001 0.2178 0.4667 60.7%

Omitting Emral (2019) 2.1050 [1.5337; 2.8892] < 0.0001 0.3294 0.5740 69.8%

Omitting Erbil (2009) 2.1090 [1.5501; 2.8696] < 0.0001 0.3144 0.5608 69.8%

Omitting Karatas (2023) 2.0571 [1.5006; 2.8198] < 0.0001 0.3246 0.5698 69.3%

Omitting Kim (2020) 2.0756 [1.5056; 2.8616] < 0.0001 0.3366 0.5801 69.4%

Omitting Lopez (2016) 2.1755 [1.5942; 2.9689] < 0.0001 0.3026 0.5501 62.4%

Omitting Moraes (2019) 2.0920 [1.5282; 2.8636] < 0.0001 0.3254 0.5704 69.8%

Omitting Rebelo (2023) 2.1123 [1.5389; 2.8994] < 0.0001 0.3289 0.5735 69.8%

Omitting Reimondo (2020) 2.2077 [1.6528; 2.9490] < 0.0001 0.2536 0.5035 66.9%

Omitting Ribeiro-Cavalari (2018) 2.0437 [1.4941; 2.7953] < 0.0001 0.3180 0.5639 68.9%

Omitting Sokmen (2018) 2.1499 [1.5868; 2.9130] < 0.0001 0.3000 0.5477 69.2%

Omitting Szychlińska (2023) 2.0419 [1.4980; 2.7832] < 0.0001 0.3118 0.5584 69.0%

Omitting Tuna (2014) 2.0332 [1.5009; 2.7544] < 0.0001 0.2992 0.5470 68.8%

Omitting Arruda (2018) 2.0169 [1.5004; 2.7110] < 0.0001 0.2807 0.5298 67.9%

Omitting Rebelo (2023) 2.0793 [1.5151; 2.8537] < 0.0001 0.3293 0.5739 69.7%

Omitting Ribeiro-Cavalari (2018) 1.9666 [1.4858; 2.6029] < 0.0001 0.2296 0.4792 64.5%

Pooled estimate 2.0918 [1.5528; 2.8179] < 0.0001 0.2981 0.5460 68.3%

**MetS**

OR 95%-CI p-value tau^2 tau I^2

Omitting Arduc (2014) 2.6459 [1.6737; 4.1828] < 0.0001 0.1833 0.4282 51.7%

Omitting Emral (2019) 3.0011 [1.8611; 4.8394] < 0.0001 0.2277 0.4772 56.8%

Omitting Erbil (2009) 2.7368 [1.8009; 4.1590] < 0.0001 0.1675 0.4093 54.9%

Omitting Karatas (2023) 2.9785 [1.8113; 4.8979] < 0.0001 0.2499 0.4999 56.8%

Omitting Moraes (2019) 2.7891 [1.7497; 4.4459] < 0.0001 0.2234 0.4726 58.4%

Omitting Rebelo (2023) 2.8268 [1.7232; 4.6372] < 0.0001 0.2552 0.5051 58.9%

Omitting Ribeiro-Cavalari (2018) 2.6829 [1.6862; 4.2688] < 0.0001 0.2021 0.4496 55.0%

Omitting Szychlińska (2023) 3.3505 [2.5306; 4.4361] < 0.0001 0.0000 0.0000 0.0%

Pooled estimate 2.8868 [1.9275; 4.3235] < 0.0001 0.1620 0.4025 52.2%

**Composite DM**

OR 95%-CI p-value tau^2 tau I^2

Omitting Akkus (2021) 2.1376 [1.7760; 2.5728] < 0.0001 0.0000 0.0005 38.5%

Omitting Araujo-Castro (2022) 2.0786 [1.7316; 2.4950] < 0.0001 0.0000 0.0020 42.7%

Omitting Dogra (2023) 1.9562 [1.6262; 2.3532] < 0.0001 0.0000 0.0009 35.3%

Omitting Erbil (2009) 2.0414 [1.6993; 2.4523] < 0.0001 0.0000 0.0000 53.6%

Omitting Kim (2020) 2.1223 [1.4894; 3.0239] < 0.0001 0.1882 0.4339 53.6%

Omitting Sokmen (2018) 2.0764 [1.7284; 2.4944] < 0.0001 0.0000 0.0003 50.0%

Omitting Reimondo (2020) 2.0743 [1.5416; 2.7913] < 0.0001 0.1108 0.3329 53.2%

Omitting Arduc (2014) 2.1335 [1.5124; 3.0096] < 0.0001 0.1738 0.4168 53.4%

Omitting Karatas (2023) 2.1430 [1.5303; 3.0009] < 0.0001 0.1627 0.4033 53.3%

Omitting Lopez (2016) 2.1398 [1.4927; 3.0674] < 0.0001 0.1944 0.4409 53.1%

Omitting Rebelo (2023) 2.0795 [1.5071; 2.8693] < 0.0001 0.1440 0.3794 53.2%

Omitting Ribeiro-Cavalari (2018) 1.9743 [1.6345; 2.3847] < 0.0001 0.0000 0.0002 50.1%

Omitting Anderwald (2013) 2.0222 [1.6845; 2.4277] < 0.0001 0.0000 0.0008 50.5%

Omitting Emral (2019) 2.0071 [1.6699; 2.4122] < 0.0001 0.0000 0.0014 50.0%

Pooled estimate 2.0438 [1.7037; 2.4518] < 0.0001 0.0000 0.0011 49.7%

**DM**

OR 95%-CI p-value tau^2 tau I^2

Omitting Akkus (2021) 1.9237 [0.8329; 4.4433] 0.1256 0.6982 0.8356 64.1%

Omitting Araujo-Castro (2022) 1.9272 [0.9077; 4.0916] 0.0877 0.5944 0.7710 67.2%

Omitting Dogra (2023) 1.2586 [0.6604; 2.3986] 0.4846 0.3113 0.5580 54.6%

Omitting Erbil (2009) 1.4637 [0.5624; 3.8092] 0.4350 1.0633 1.0312 74.8%

Omitting Kim (2020) 1.4422 [0.5174; 4.0201] 0.4838 1.1692 1.0813 74.6%

Omitting Sokmen (2018) 1.7365 [0.7030; 4.2892] 0.2316 0.9181 0.9582 72.6%

Omitting Reimondo (2020) 1.3980 [0.5262; 3.7144] 0.5015 1.0738 1.0363 74.2%

Pooled estimate 1.5748 [0.7012; 3.5367] 0.2713 0.8139 0.9021 69.8%

**DL**

OR 95%-CI p-value tau^2 tau I^2

Omitting Araujo-Castro (2022) 1.2562 [0.9684; 1.6294] 0.0857 0.0643 0.2536 43.0%

Omitting Arduc (2014) 1.2839 [0.9836; 1.6760] 0.0661 0.0595 0.2440 39.9%

Omitting Emral (2019) 1.3324 [1.1197; 1.5856] 0.0012 0.0000 0.0020 16.2%

Omitting Karatas (2023) 1.1698 [0.8884; 1.5403] 0.2641 0.0722 0.2687 41.3%

Omitting Kim (2020) 1.1819 [0.8593; 1.6257] 0.3041 0.1124 0.3353 45.4%

Omitting Lopez (2016) 1.1915 [0.8582; 1.6542] 0.2953 0.1210 0.3479 46.2%

Omitting Moraes (2019) 1.2084 [0.9135; 1.5984] 0.1849 0.0843 0.2903 46.2%

Omitting Rebelo (2023) 1.1684 [0.9047; 1.5090] 0.2331 0.0543 0.2331 37.4%

Omitting Reimondo (2020) 1.2395 [0.9494; 1.6183] 0.1145 0.0721 0.2685 45.3%

Omitting Ribeiro-Cavalari (2018) 1.1674 [0.8934; 1.5254] 0.2569 0.0642 0.2534 39.7%

Omitting Szychlińska (2023) 1.2652 [0.9900; 1.6169] 0.0602 0.0520 0.2280 36.9%

Pooled estimate 1.2269 [0.9514; 1.5821] 0.1150 0.0639 0.2529 40.4%

**CVE**

OR 95%-CI p-value tau^2 tau I^2

Omitting Araujo-Castro (2022) 1.3142 [0.6611; 2.6123] 0.4357 0.2423 0.4922 52.2%

Omitting Dogra (2023) 0.9660 [0.6933; 1.3459] 0.8381 0.0000 0.0014 8.0%

Omitting Kim (2020) 1.0575 [0.6588; 1.6977] 0.8168 0.0617 0.2484 24.3%

Omitting Lopez (2016) 1.5317 [0.7411; 3.1654] 0.2497 0.1374 0.3706 28.0%

Omitting Reimondo (2020) 1.3865 [0.7467; 2.5743] 0.3007 0.1809 0.4253 45.0%

Pooled estimate 1.2193 [0.7149; 2.0796] 0.4667 0.1297 0.3601 36.3
